# The cognitive neuroscience and neurocognitive rehabilitation of dance

**DOI:** 10.1186/s12868-024-00906-8

**Published:** 2024-11-06

**Authors:** Madeleine Eve Hackney, Agnieszka Zofia Burzynska, Lena H. Ting

**Affiliations:** 1 Center for Visual and Neurocognitive Rehabilitation, Atlanta VA HealthCare System, 1670 Clairmont Road, Decatur, GA 30033 United States of America; 2 Emory University School of Medicine Department of Medicine, Division of Geriatrics and Gerontology, 100 Woodruff Circle, Atlanta, GA 30322 United States of America; 3 Department of Veterans Affairs Birmingham, Atlanta Geriatric Research Education and Clinical Center, 3101 Clairmont Road, Brookhaven, GA 30319 United States of America; 4 Department of Rehabilitation Medicine, Division of Physical Therapy, Emory University, 100 Woodruff Circle, Atlanta, GA 30322 United States of America; 5 Department of Human Development and Family Studies, Colorado State University, 303 Behavioral Sciences Building, 410 W. Pitkin St., 1570 Campus Delivery, Fort Collins, CO 80523-1570 United States of America; 6 Wallace H. Coulter Department of Biomedical Engineering, Emory University and The Georgia Institute of Technology, 1760 Haygood Drive, Suite W 200, Atlanta, GA 30322 United States of America; 7 57 Executive Park S, #219, Atlanta, GA 30329 United States of America

**Keywords:** Dance, Cognition, Mental imagery, Sensorimotor integration, Neural representation, Dance therapy, Rehabilitation, Partner, Groove, Synchrony, Robotics, Neurodegeneration

## Abstract

Creative movement, in the form of music- and dance-based exercise and rehabilitation, can serve as a model for learning and memory, visuospatial orientation, mental imagery, and multimodal sensory-motor integration. This review summarizes the advancement in cognitive neuroscience aimed at determining cognitive processes and brain structural and functional correlates involved in dance or creative movement, as well as the cognitive processes which accompany such activities. We synthesize the evidence for the use of cognitive, motor, and cognitive-motor function in dance as well as dance’s potential application in neurological therapy and neurorehabilitation. Finally, we discuss how partnered interaction and sensorimotor integration in dance, and “dancing robots” could shed light on future application of dance as rehabilitation, of dance used in technology and potential mechanisms of benefit from dance-based activities.

## Introduction

Dance is a form of creative movement that has been a key aspect of human culture and experience for at least 10,000 years [1]. As a complex behavior, in addition to its motor aspect, dance integrates and relies on multiple cognitive functions, including memory, attention, executive functions, psychomotor speed as well as social cognition and emotions [2]. This use of multiple cognitive functions makes dance a unique model for studying learning and memory, neural representation, mental imagery, interpersonal synchrony, and/or multimodal sensorimotor integration. Some researchers are actively engaged in learning how dance practice molds the brain over time and through different regimens [3, 4]. Clinical researchers are also keenly interested in the utility of dance in cognitive or motor therapy and rehabilitation [5], and supporting brain health in advanced age [6]. Cutting edge research has revealed movement principles across motor skill and rehabilitation [7]. These principles can be applied to our understanding of dance as an activity engaged in through a lifetime, and for its application to technology and robotics in various human-robot scenarios. In this synopsis, we provide examples of creative movement paradigms that can be investigated for neural, cognitive and rehabilitative utility, such as partner interaction, sensorimotor integration, and “dancing” robots.

In this review, we discuss how advances in human neurosciences in the past three decades, especially related to non-invasive functional and structural imaging of the human brain, have facilitated burgeoning research on the cognitive and neural processes involved in creation and performance of dance. As dance has been shown to be a promising therapeutic tool in various patient populations with cognitive or neural disorders, we also review the emerging evidence for cognitive and brain correlates of dance practice in healthy populations and in rehabilitation settings. Finally, we discuss how cognitive neuroscience of dance could inform harnessing the technology (e.g., artificial intelligence (AI)) for future rehabilitation opportunities using human-robot interactions. In sum, this review investigates the utility of dance in advancing basic understanding of human cognition and brain function, as well as using dance to support or enhance neural health and cognitive performance. In the next sections, we first define key concepts for this review, such as dance, cognitive function, and tools used in cognitive neuroscience, after which we review the existing research on cognitive neuroscience of dance, and outline directions for future investigations into dance and its application for health.

### What is dance?

Defining dance is challenging due to its breadth and uniqueness. Broadly, dance can be defined as a form of expressive or creative movement, often but not always performed to music, that involves coordinated, rhythmic actions of the body. Yet, dance, which represents the translation of creative cognition into action, is distinct from other form of movement such as yoga or gymnastics. Dance should be defined through the goals of a given context and environment. Emotional expression can be an element found in dance [8]. Some researchers define dance as “consciously organized energy that gives form to feeling” [9]- which speaks to the affective, emotive qualities of dance. Dance can be used to tell stories, to further the goals of political regimes or to be an exploration of movement. Emotional and aesthetic elements are intertwined with the physical act of dancing, creating a unique mode of non-verbal communication within and across cultures.

Choreography may be danced solo, partnered or in groups. Solo dance emphasizes individual movement and is represented, for example, in ballet, contemporary and theater dance. Partnered dance involves coordinated movements between two people, as seen in tango, ballroom dance, or salsa, where synchronization and mutual responsiveness are necessary. These types of dances rely heavily on social cognition and shared motor intentions [10]. Group dances, such as traditional folk dances or synchronized performances, emphasize collective movement and social bonding through shared rhythm and timing. Across all dance types, fundamental movement principles govern the execution of movements, including balance, weight transfer, rhythm, and spatial awareness [11]. These principles are not only essential for performing the physical aspects of dance but also for interpreting and responding to movement, whether in a social or performative setting. Dance therefore encompasses social, aesthetic, physical and cognitively engaging spheres that make it a ripe movement form for scientific inquiry, and particularly through cognitive neuroscientific methods.

### Cognitive neuroscience and its tools

Cognitive neuroscience is a multidisciplinary field that studies the neural mechanisms underlying cognitive functions, integrating methods and concepts from neuroscience and psychology. The field aims to understand how brain structure and activity is linked to cognitive processes including attention [12], memory (short-term, working, and long-term memory) [13], executive function [14], language [15], visuospatial skills [16], processing speed [17], and social cognition [18].

The field of cognitive neuroscience has flourished thanks to the development of non-invasive neuroimaging techniques, magnetic resonance imaging (MRI), as well as electro encephalography (EEG), positron emission tomography (PET), and magnetoencephalography (MEG), that allow measurement of brain function and structure in living humans. In the context of studying dance, the key technique has been MRI. This includes structural MRI, which assesses macroscopic brain features such as volumes of the grey and white matter, cortical thickness, or volume of white matter lesions or hyperintensities, functional MRI (fMRI), which relies on the blood oxygen level dependent (BOLD) signal to detect changes in neural activity during a task or at rest, and diffusion MRI, which allows inference about tissue microstructure and structural connectivity from directionality and magnitude of diffusion of water protons within highly organized tissues, such as the myelinated axonal bundles in the white matter. The field also uses standard, clinical neuropsychological exams of multiple different cognitive domains.

## Cognitive processes involved in dance

Various cognitive processes are hypothesized to be involved in and trained by dance practice. These cognitive processes are necessary for accomplishing dance movements and to engage with others in group or partnered dance.

Dance typically involves storing new steps and recalling previously learned steps in a sequence or improvisational pattern, learning new steps or moves and practicing them immediately, which involves sequential memory, spatial working memory, relational memory, or broadly defined non-declarative long-term memory. In addition, partnered dance participants walk in step patterns with intricate and often changing spatial relationships between the two partners, which may enhance encoding spatial information to memory [19]. Consistent with this idea, early studies have shown that professional ballet dancers show better performance on motor recall for structured choreography than novice participants [20]. A similar study on modern dance also showed superior choreography performance in expert dancers, in both structured and unstructured scenarios, suggesting that superior memory performance in dancers may go beyond domain-specific knowledge of dance structure as known in classical ballet [21]. More recently, a study comparing capable professional dancers with age-, sex- and education-matched novices showed that although dancers outperformed novices on balance task and real-time dance mimicry in the Dance Central 2 (Harmonix Music Systems 2011) video game, the two groups did not differ on laboratory tasks of sequential memory, spatial working memory, and relational memory [4]. These results suggest that the benefit of dance training may be specific to dance-related memory and may be most evident when using motor recall in the presence of music (i.e., memory supported by musical beat detection and interpretation), as suggested in the earlier studies [21]. Possibly, general memory benefits of dance are not yet evident in healthy young adults undergoing college education, and that protective effects of dance on cognition could increase with advancing age. This possibility agrees with epidemiological studies reporting that people with a life-long history of dancing are less likely to be diagnosed with dementia or to experience age-related cognitive decline [22]. Laboratory cognitive tasks (computer or paper-and-pencil-based) may not be able to capture the effects of dance training on memory. Together, it is necessary to develop cognitive tasks that better capture memory processes trained by dance and their utility in every-day life and for comparisons of dancers with novices across the lifespan and in different training and cognitive health contexts.

Attention also seems to play a central role in dance, as participants need to divide attention between postural control, the current pattern and trajectory, music/beat, and partner cues, all of which challenge balance and cognition [23]. Executive function is required for planning and organization of steps, inhibition and task switching needed to leave one step or rhythm and begin another, or to respond to an unexpected cue from a partner [24]. Visuospatial cognition is also expected to benefit from dance training given its central role in dance movements and the fact that cardiovascular fitness, also involved in dance, modulates brain activation associated with spatial learning [25].

Sensory-motor integration goal-communicative structure of partnered and group dances requires additional cognitive processes related to interpersonal communication and social cognition [26]. For example, partnered/group dance requires coordinating body movement with both an external musical source and the partner(s) [26], and stringing together steps into phrases, holding sensorimotor ‘conversations’ with the partner(s) via non-verbal communication [27]. In addition, partnered/group dance puts additional demand on spatial cognition as it requires processing coordinated relationships between people, places, and objects, as well as sustained attention to others to coordinate the next step and navigate the space safely [28].

## Neural correlates of dance

Although the cognitive benefits of dance training seem elusive, neuroimaging methods have enabled identification of brain regions that are activated while performing dance or processing dance information. An earlier review covered cognitive neuroscience research on dance until 2015 [29]. As such, we briefly summarize its key points and extend it with newer studies.

### Brain activity

Leaning choreography typically involves watching others perform choreography and practicing one’s movement while gazing intermittently in a mirror. Therefore, the action observation network (AON), a set of brain regions activated when watching others perform an action and crucial for planning, coordinating, and executing movements, has been used as a paradigm for studying brain function related to dance. Brain activations within the AON (i.e., premotor, intraparietal, posterior superior temporal and parietal cortices, as well as in medial prefrontal, cingulate, and parahippocampal gyri) were found to be significantly greater while viewing familiar movements compared to unfamiliar movements in expert capoeira Dancers [30] and in expert versatile dancers than in novices when watching videos of modern dance [4, 31]. Moreover, activation of the AON in dancers was positively associated with participants’ dance experience and performance [32] as well as accuracy on the Dance Central video game [4]. Thus, expert dancers can activate the AON on demand to a greater extent, which is associated with superior dance performance.

### Brain structural connectivity

Another indication of brain specialization for a given skill may be alteration in strength of co-activation of specific brain regions measured using fMRI or EEG, known as functional connectivity (FC). We have shown that expert dancers differed on resting state FC within the motor learning system, which was related to both increased and decreased FC [4]. These differences in connectivity were consistently correlated with performance on Dance Central video game (aka the dance skill). Increased resting-state FC in expert modern dancers vs. novices were also found within the sensorimotor network and cortico-basal ganglia loops, controlling body posture, movement, and action selection [33]. These fMRI studies, however, compared resting state FC in expert dancers and novices out of the dance context. Thus, important evidence of dance processing was provided by an fMRI study in which dance novices watched dance videos. This study showed increased FC in several networks relevant for cognition such as the attention and frontoparietal control, as well as visuospatial, somatosensory, body motion, motor, visual, and complex sound processing [34]. As performing full choreography inside an MRI scanner is not possible, important insights can be provided by EEG, despite its lower spatial resolution and source specificity, compared to fMRI. An EEG study assessed brain activity and FC during physical execution and while imagining choreography, with or without music, in dance-inexperienced women after three sessions of modern jazz dance. Despite the short length of training, low level of proficiency, and lack of a control group, this promising feasibility study showed increases in FC in many frequency bands during dance execution, that were modulated by the presence of music [35]

### Brain volume

Alterations in brain structure associated with dance expertise has been studied by comparing experts with novices. A comparison of low-body mass index (BMI) professional ballet dancers and novices with normal BMI revealed decreased volumes of the premotor and supplementary motor cortices, in the putamen, and the corticospinal tract in dancers [36], although a comparison of age-, sex-, education- and BMI-matched versatile dancers and novices revealed no differences in cortical thickness or volume [4]. Other studies showed localized increases in cortical volume in expert dancers: an increased brain volume was found in the motor area of the foot in professional ballet dancers as compared with handball players [37] and both dancers and musicians, as compared to novices, showed increased superior temporal cortex thickness [38]. Iin all, the findings on brain structure alterations related to dance expertise are inconclusive, given that increases and decreases in brain volume have been observed in motor and cognitive training [39]. The existing findings need to be revisited in larger samples, using a more detailed definition of dance expertise and specialization. Higher resolution and more advanced MRI methods may give more insights into expertise-related brain modifications

### White matter microstructure

The most widely used diffusion MRI technique for studying white matter microstructure in the past two decades has been diffusion tensor imaging (DTI). The derived measure, called fractional anisotropy (FA), reflecting a greater level of microstructural organization related to myelinated axons, has been shown to decline in aging and disease [40, 41]. However, dance expertise in young participants has been consistently linked to decreased FA, for example, in the corpus callosum, premotor, and lateral prefrontal white matter in ballet dancers [36], in the ventral prefrontal white matter, corticospinal tract, and the superior longitudinal fasciculus in versatile expert dancers [42], and in the corticospinal tract of versatile expert dancers [4]. Interestingly, lower FA in the corticospinal tract correlated with better Dance Central game performance and with FC [4], suggesting that the reports of lower FA could be associated with structural adaptations linked to functional networks and superior dance performance.

## Affective cognition in dance

Dance has been shown to involve and regulate emotions and mood. fMRI studies have shown that dance activates brain areas associated with reward, such as the ventral striatum and orbitofrontal cortex, related to mood regulation [2, 43–45] as well as the amygdala and insula [8, 10, 46], which are responsible for processing emotional stimuli and feelings [2]. Dance is also a pleasurable experience due to its aesthetic component. For example, when individuals watch or engage in dance, especially when they find it aesthetically pleasing, brain regions related to reward system (ventral striatum and orbitofrontal cortex) have been shown to be activated [32]. Synchronized group dance has been shown to elicit brain activation in regions associated with empathy and social cognition, such as the medial prefrontal cortex and anterior cingulate cortex [45]. Dance, especially in group settings, is considered to play an important role in enhancing empathy and social bonding. Some research has shown that synchronized movement, which occurs in social dances, fosters a sense of connectedness and improves emotional attunement between participants [10]. For this reason, dance/movement therapy (D/MT) has been applied to populations with various mental health conditions, including post-traumatic stress disorder (PTSD), depression, and anxiety, with promising results. DMT integrates body movement with emotional processing, helping individuals express emotions that may be difficult to articulate verbally. Studies show that DMT can lead to decreased cortisol levels and enhanced mood, suggesting that dance can regulate stress and emotional distress. Similarly, dance interventions have been associated with significant reductions in depression and anxiety symptoms, often outperforming other forms of physical activity due to the emotional expression and social interaction involved [43]. Through movement and expression, dance can evoke strong emotional responses, which are processed by the same networks that respond to other rewarding stimuli. Thus, dance is a promising tool for promoting emotional regulation, social bonding, and therapeutic intervention, which urges more research on neural correlates of affective aspects of dance.

## Dance as a tool for supporting cognitive and brain health in aging

Normative brain aging is associated with changes that can be measured using MRI, such as reduced cortical thickness [47], reduction of hippocampal volume [48, 49], appearance of white matter hyperintensities and reduced microstructural white matter integrity as measured with DTI [41, 48, 50], as well as reduced density of myelin and axons measured with more advanced MRI [51–53]. Alzheimer’s disease (AD) and other neurodegenerative diseases which affect millions of people world-wide are characterized with even more dramatic structural changes in both grey and white matter [54]. One of the best known and evidence-based lifestyle factors that protect brain health across ages is regular physical activity and exercise [48, 55–57]. Many people struggle to maintain a physical activity routine, which can become more difficult with advanced age and increasing health limitations [58]. Dance as a form of physical and social activity is popular among older adults in various parts of the world [59]. Dance involves elements of novelty, fun, and social interaction, that may encourage regular, long-term participation. Professional dancers are known to better maintain motor and cognitive skills into old age [22]. A recent systematic review on randomized controlled trials in healthy older adults or in people with mild cognitive impairment (MCI) concluded that dance training led to consistent positive structural and/or functional changes in the brain [60] Structural changes included increased hippocampal volume, gray matter volume in the left precentral and parahippocampal gyrus, concomitant with significant improvement in memory, attention, body balance, psychosocial parameters and altered peripheral neurotrophic factor [61]. These effects of dance training clearly present a promising strategy to slow down or even reverse the cognitive and neural processes associated with normative and possibly, neuropathological aging. Recent evidence suggests robust within-person declines in the white matter microstructure over time periods of months to years, as measured with DTI. These declines accelerate in older age and in the presence of risk factors such as lack of physical activity or cardiovascular disease [62]. Yet, 6-months of dance training was significantly linked to a reduced deterioration in the vulnerable prefrontal white matter [57] and increases in the FA in the fornix [6], a fiber bundle connecting the hippocampus whose deterioration predicts a transition from MCI to dementia [62]. As such, dance training as a form of exercise helped provide some of the first evidence for plasticity or recovery in the aging white matter using non-invasive, in vivo imaging methods. This discovery opens the possibility of restoring white matter health using dance which is non-pharmacological, widely accessible, low cost, side effect-free, and socially satisfying. This finding is of particular importance for two reasons: (1) the accessibility of dance may help resolve disparities in brain health in marginalized and underserved groups, both as a preventive and intervention strategy, and (2) targeting white matter pathology through dance in aging and dementia could open new avenues for treatment, given that available treatments targeting amyloid and tau pathology in the grey matter have shown limited effectiveness combined with high cost and risk of side effects [63].

## Dance as a tool for neurocognitive and motor rehabilitation in neurodegenerative and neurological disease

The last decade or so has produced evidence supporting the use of dance in neurorehabilitation in chronic neurological diseases such as spinal cord injury, AD, Parkinson’s disease (PD), multiple sclerosis, chronic pain, cerebral palsy, severe and persistent mental illness (e.g., PTSD, depression), among other conditions [5]. These benefits may have resulted because dance combines physical with cognitive learning/training, inducing beneficial, lasting effects on the brain’s structure and function [64]. As mentioned in the sections before, some perceived advantages of dance used as treatment for many patients stems from the combined experience of learning to move to music and rhythms while socially interacting, which likely taps into theories of neural plasticity for gaining and losing function. Many dances require some form of adaptation for a given patient population because the goals of dance used as treatment differ from dance used for pure social, dance exercise, or virtuosic performance reasons. Growing evidence suggests that participation in partnered dance for example, can induce long-term neuroplasticity in human movement, enhance independence, and delay the deleterious effects of aging and neurodegenerative diseases; however, the precise neuroanatomical mechanisms underlying these benefits remain unknown. The goals of dance used therapeutically include enhancing behavioral cognitive, motor- and emotional function; however, there is also great interest in whether dance rehabilitation can positively impact the underlying neuropathophysiology of disease and/or the efficacy of pharmacological treatments used to treat the conditions.

### Adaptango

As some aspects of dance used as treatment have been covered in a recent review [65], here, we focus on a specific example of adaptation of a dance, Argentine tango dance (Adaptango), for partnered rehabilitation in various patient groups [66, 67]. The dancing pair in Adaptango maintain an embrace, also known as a “frame,” which is a fixed arm position linking the two dancers. This physical connection enables a sophisticated, yet accessible tactile communication system that conveys motor intentions and goals between dancers. Both dancers must maintain internal focus while also cognitively attuning to the environment and other individuals, including their partner. While tango dance draws on a core vocabulary of steps (e.g., *ochos* (figure eights) and *cruzadas* (crosses)), dancers improvise the order and execution of the steps in social dancing. Leading and following roles in Adaptango offer the potential for effective therapy that engages learning to address motor, cognitive and psychosocial impairments.

Adaptango training has been linked to significant gains in mobility, balance, and health-related quality of life [68–71], endurance, cognition, spatial cognition, reduced disease symptom severity [19], and increased participation, the World Health Organization’s construct [72] for the ability to engage in life’s activities [73, 74]. These improvements may persist from one [68] up to three months after ending Adaptango treatment [19, 75, 76]. Dissemination of Adaptango methods and pedagogy with fidelity is possible as demonstrated by a study that showed the 12-week Adaptango program could be implemented and offered in the community by several newly trained instructors [19]. Dance may have an immediate effect on mobility in those with PD as improvements have been found in as little as 2 weeks of daily Adaptango sessions [73], and participants could sustain also high volume, daily Adaptango over 3 weeks [77].

Other older adult populations, e.g., those with low vision [78] and oldest-old adults [79], have benefitted from a very similar Adaptango program as that designed for those with PD. The effects of Adaptango as treatment for people with MCI will be revealed upon completion of randomized controlled trials [5].

Importantly, Adaptango may be a health-promoting behavioral intervention that may benefit inflammatory profiles and cognition in older Black/African Americans (B/AA), who are at higher risk of developing dementia because of parental history of AD and related dementias (ADRD). This population may benefit from rehabilitative strategies that prevent or delay development of ADRD [80], e.g., an intervention that reduces inflammatory markers. In B/AA women at risk for dementia, we conducted a phase I randomized controlled trial to assess the impact of a 12 week, 20-lesson adapted Adaptango intervention (*n* = 24) to a usual care control group (*n* = 10) on measures of plasma inflammatory markers, cognition, motor and psychosocial performance. After treatment, Tango participants had significantly decreased inflammatory cytokines, including reductions in interleukin (IL)-7, interferon-gamma (IFN-γ), tumor necrosis factor-alpha (TNFα), and monocyte chemoattractant protein-1 (MCP-1) compared to those who participated in usual care (control). Reduced inflammatory load could have implications for chances of developing AD in the future, or for delaying an inevitable diagnosis. Large effects were also noted for the Tango group on tests of executive functioning and inhibition. These gains were attained for this vulnerable group through a non-pharmacologic, affordable partnered dance intervention [81].

### Using tactile information in partnered dance to communicate movement spatial and temporal goals

Among dances worldwide, partnered dance, which involves a leader and follower, stands out because it is a cognitively demanding, mentally stimulating *movement conversation* that anyone can learn to do. Culturally, and historically, individuals have chosen to dance either the leader or follower role in partnered dance, usually as per gender role. When following in partnered dance, participants cognitively engage to focus on external cues, which access neural pathways related to motor-cognitive integration [66, 67] which may be disrupted in conditions like PD.

In Adaptango, participants use one of two motor training approaches: (a) leading, consisting of primarily internally guiding (IG) motor plans, i.e., determining direction, timing and amplitude of steps and (b) following, i.e., responding to external tactile (and sometimes visual) guidance (EG). Coordinating movement with external cues demands continuous postural, visual, positional and auditory state monitoring. Adaptango dancing is improvisational, (i.e., step patterns are not choreographed), steps are often determined by the leader from many possible steps and the follower does not know what the next step will be. Thus, tactile information serves as a conduit for motor-cognitive processes in human-human interaction (HHI) necessary in partnered dance. The importance of tactile information in the HHI of dance becomes clear in that both leading and following can be achieved even with eyes closed [82]. Physical interactions between two people can provide a motor manipulation through tactile stimulation, or haptic feedback. The HHI practiced through partnered dance primarily acts as communication [83], which is also capable of altering motor behavior (e.g., gait spatiotemporal characteristics, and challenging, or stabilizing, balance).

The neural networks underlying self-initiated movement and cued movement are distinct [66] and thus, leading and following use different neural and cognitive mechanisms to accomplish the roles. When following in partnered dance, participants focus on external cues, which access alternate neural pathways including the cerebellar-thalamo-cortical (CTC) network [66, 67]. In healthy adults, external cueing recruits sensory response networks interposed with motor execution regions operating in a feedback and match to target mode. These motor execution regions include the cerebellum for titration of movement of the lower extremity and cortico-cerebellar pathways to facilitate conscious control [78]. Conditions like PD may benefit from external cueing while using the CTC pathway for example, which may be augmented by partnered dance training. As such, in partnered dance, the leader plans the step length, rotation, timing and direction of the dyadic unit. Then from the external cues, the follower infers and enacts the motor intentions of the leader, which our work has shown can be communicated solely through force and pressure at contact points between the two [84].

As mentioned before, highly relevant to partnered dance when delivered as rehabilitation is the *tactile cueing* necessary for the dance communication. Some research has shown that tactile cues are processed faster, with less attentional demand, and more efficiently than visual and auditory cueing. Somatosensory integration mechanisms for prioritizing tactile and proprioceptive feedback have fortunately been shown to not be disrupted by neurodegenerative disease, e.g., PD [85]. Further, somatosensory cueing can supersede visual distractors [86]. Using haptic speed cues from a moving handrail, people with PD walked faster by spontaneously (i.e., without specific instruction) increasing stride length without altering cadence [87]. Tactile cues (delivered via a smartphone) have decreased timing errors during a dual task scenario [88]. Rhythmic somatosensory cues are helpful in increasing turning speed and may be more effective than visual cues [89]. Furthermore, humans can abstract metric structure (pattern of beats) from tactile rhythms as efficiently as from identical auditory patterns [90]. Followers in partnered dance often abstract the stepping pattern from the leader in this tactile fashion, when receiving and responding to the step timing information conveyed with touch cues by the leader. Responding to tactile cues in a partnered dance context should be explored for potential rehabilitative purposes.

Anyone who has attempted dance may agree that dance programs challenge participants to learn unfamiliar movement combinations, hold several steps and directions in their memory, and attend to multisensory cues, all while aiming for a certain pose of the body and generally while moving to an external auditory beat. Studies should further investigate the precise amount and timing of mentally stimulating, cognitively engaging, and potentially neuroprotective properties of neurocognitive rehabilitation like partnered dance.

## Dance and robotics: the next frontier

Movement experiences over a lifetime affect our motor repertoire [91] posing a challenge for understanding the principles of the diversity in movement across individuals [92]. How people move their bodies and cooperate with others in various forms of dance provides insight into fundamental principles of how individuals move across the spectrum from motor skill to motor impairment [91, 93] and, therefore, could be applied to technology and robotics in various human-robot scenarios, including rehabilitation. Here, we provide examples of creative movement paradigms that can be investigated for partner interaction, sensorimotor integration, and “dancing” robots.

### Dance research can reveal movement principles across motor skill and rehabilitation

We have leveraged the specific training involved in different dance forms to test theories about how training and experience alter how individuals move across the spectrum from motor skill to motor impairment [93]. Movement experiences over a lifetime affect how we move [91, 92] posing a challenge for understanding the principles of the diversity in movement across individuals. Aspects of motor skill learning in dance may transfer to other challenging balance tasks and shape how we walk in everyday life. We hypothesize that muscle coordination patterns–called motor modules or muscle synergies–are learned building blocks of movement that give rise to “motor accents”, or individual styles in movement. We showed that pre-professional and professional dancers use similar motor modules in a challenging beam traversing task [94] and in normal overground walking whereas non-dancers did not [83]. Thus, motor skill learning as in dance appears to shape existing movement patterns for everyday tasks and may also expand the capabilities of those patterns to accomplish more challenging motor tasks. Not only could generalizability of motor modules tasks play a role in why we may be recognized based on our gait, it also informs how dance-based and other rehabilitation may generalize to real-world situations [92]. After participation in Adaptango, we showed balance improved [77] and motor modules became more consistent in individuals with PD [3].

As we learn motor skills, one’s control becomes more automated, enabling the brain’s cortical resources to be engaged in higher level functions such as navigation and memory, or other concurrent cognitive and motor tasks. Across levels of motor skill, we showed that dancers demonstrated the most similarity in motor modules for walking and involuntary balance correction, whereas individuals with PD and stroke survivor showed the greatest dissimilarity in proportion to the level of movement impairment [3, 95, 96]. We hypothesized that greater engagement of cortical resources is required for movement when an individual suffers motor impairments. To address this question more directly, we began direct recording of brain activity elicited during balance recovery following a sudden movement of the support surface. Even in young adults, we demonstrated that those who performed poorly on a beam-walking task also had larger cortical responses in balance recovery [7]. Similarly, older adults with greater evoked cortical responses also had poorer clinical balance and balance confidence scores [7, 97]. We predict that dance-based rehabilitation can reverse these trends.

### The robots are coming

We also use HHI/partner dance as a framework for understanding communication through physical interaction that can inform the design of assistive and rehabilitation robots. Robotics are gaining immense attention, because of their potential to reach multiple populations in rehabilitative scenarios. Our goal is to identify principles of this motor coordination and use them to design easy to use and intuitive assistive robotics, particularly ones that accompany you under their own power, such as a robotic walker. Partner dance involves communication and adaptation of movement through physical interactions. Surprisingly low forces (< 30 N) are used by participants to synchronize stepping between partners, with lower forces when two novices are partnered [98]. Similarly low forces levels are used during walking assistance through handholding, demonstrating that much of the benefit is through haptic communication rather than physical assistance [99]. We have attempted to replicate aspects of the interactions in physical human-robot interaction at the hands during walking [84, 100], and can alter gait parameters [99].

Human-robot partnered dance is an ideal scenario for testing hypotheses about motor goal communication between robots and humans. Gaining knowledge of the subjective experience of humans with robots in rehabilitation is vital to inform and enhance the technology for treatment delivery. In a series of HHI studies, we focused on communications through the hands during the partnered dance experience, and how it can affect step initiation, direction, amplitude, and timing. We have shown that in investigations of leading and following in human-robot interaction scenarios, touch information alone was sufficient to perform partnered dance with complex movements, like what social dancers learn in their classes [101]. In our study, Chen et al., (2015) [84] demonstrated that a robot could be programmed to respond to (i.e., follow) touch cues given by a blinded, expert human-leader that indicated the timing, amplitude and direction of steps. The robot was able to follow the human-leader with a lag of 224 ± 194 ms. using only forces at the hand. Human leaders reported that dancing with the robot was like dancing with a human. We measured the objective and subjective measures of the physical human-robot interactions when robot arm stiffness and the gain of the robot’s base velocity with respect to the hand forces were varied. We developed and validated a questionnaire to evaluate the subjective experience and perceptions of the human partners. The magnitude of interaction force, cadence, lag, and distance between leader and follower changed across conditions and were correlated to the subjective experience of the humans (e.g., was the robot a good dance partner? Was the experience like dancing with a human? ). Expert dancers rated their physical interaction with the robot more favorably when biomechanical metrics of synchrony between the human and the robot were greatest and when interaction forces were lowest. We continued this research with older adults, determining the acceptability of robots used in a dance scenario for several older adult participants [100]. While these studies represent just the first steps, dance and dance-based rehabilitation provide a great opportunity to understand how we move, the thought processes underlying movements and how we can help people retain mobility and function throughout their lives. Robotics and Artificial Intelligence will undoubtedly play an important role across rehabilitative modalities, including those which are dance-based.

## Conclusions

In sum, research on cognitive neuroscience and neurocognitive rehabilitation of dance highlight the complexity of dance as a motor, cognitive, emotional, and social experience. Dance engages broad networks in the brain, including executive function, motor planning areas, sensory processing regions, emotional centers, and social cognition networks. Future research may explore how different dance styles and cultural contexts shape these neural representations. Growing evidence suggests that participation in dance may support brain and cognitive functioning, enhance everyday independence and quality of life in clinical populations, and delay the deleterious effects of aging and neurodegenerative diseases. While strides have been made in understanding dance’s role in cognitive processes, neurocognitive rehabilitation, its utility in understanding HHI, and its future in technology, several key questions remain to be addressed in future studies, such as (a) understanding the timing and neural mechanisms involved in dance learning, (b) harnessing advanced MRI [102] to understand how dance “molds” the brain, (c) determining brain activity patterns during dance performance, and how they evolve at different levels of expertise, (d) comparison of dance to other forms of cognitive or physical training (e.g. [5]), combining neuroimaging with blood biomarkers, vascular function, motion analysis, and other mechanistic approaches to study dance for its rehabilitative and preventative potential.

The opportunities to explore the cognitive neuroscience of dance are diverse in methodology, assessment, theoretical underpinnings, and types of intervention. More research is needed to drive the field forward in creative ways. The studies presented here have limitations and biases. Along with neuroimaging, and blood biomarkers [81], dance as rehabilitation could be examined for its synergistic effects with pharmacological and surgical regimens. As such, dance research needs scrutiny and must adhere to principles of rigor in neuroscience, neurocognitive and neurorehabilitation research, which is recognized by e.g., the field of music [26], and needs to be formally stated in dance. With teams working in partnership, major advances should occur in the next decade for understanding of neurological and neuroscientific mechanisms underlying dance-based approaches.

## Data Availability

No datasets were generated or analysed during the current study.

## Declarations

### Declarations

This paper concerns a review of previously published work.
